# Identifying Micropapillary Patterns With Fine-Needle Aspiration Biopsy at Metastatic Sites to Enhance Breast Cancer Diagnosis: A Case Series

**DOI:** 10.7759/cureus.100075

**Published:** 2025-12-25

**Authors:** Ojas Gupta, Ram Nawal Rao, Rahul Gupta, Gaurav Agarwal

**Affiliations:** 1 Pathology, Sanjay Gandhi Postgraduate Institute of Medical Sciences, Lucknow, IND; 2 Endocrine and Breast Surgery, Sanjay Gandhi Postgraduate Institute of Medical Sciences, Lucknow, IND

**Keywords:** breast cancer, cytology, fine needle aspiration biopsy, fnab, impc, invasive micropapillary carcinoma, male breast cancer, metastasis, micropapillary, mucinous carcinoma

## Abstract

Invasive micropapillary carcinoma (IMPC) of the breast is a rare and aggressive subtype of invasive breast carcinoma, distinguished by its unique morphological characteristics. This case series analyzes fine-needle aspiration biopsy (FNAB) specimens obtained from metastatic sites in suspected breast cancer patients between 2022 and 2024, integrating detailed clinical, histopathological, and immunohistochemical information. Six samples (four axillary lymph nodes, two skin nodules) from four patients (three females, one male; mean age 60) were analyzed. Cytological examination revealed variability in cellularity and grade, as well as the presence of angulated, avascular papillaroid and morule/cell ball-like clusters with reversed cellular polarity and variable background mucin. Notably, increased cellularity, atypia, and distinct cytological patterns at metastatic sites on FNAB, coupled with reduced background mucin, showed a strong correlation with a higher proportion of IMPC in the corresponding primary breast tumors. FNAB emerges as a rapid, minimally invasive, and cost-effective technique for assessing peripheral metastases, aiding not only in the detection of this rare and aggressive subtype but also in guiding clinical management by providing valuable insight into its extent within the primary tumor.

## Introduction

Invasive micropapillary carcinoma (IMPC) of the breast is an uncommon histological subtype of invasive breast carcinoma (IBC), representing approximately 0.9-2% of cases [1]. Clinically, IMPC exhibits highly aggressive behavior, characterized by extensive lymphovascular invasion (LVI) and frequent regional lymph node metastasis (LNM), rendering it more malignant than conventional IBC [2].

WHO has defined diagnostic criteria of IMPC that require the presence of neoplastic cells arranged in micropapillary or mulberry-like clusters lacking a fibrovascular core on histopathology, along with reversal of cell polarity. This reversal is demonstrated both by histomorphology and by the inside-out expression of epithelial membrane antigen (EMA) on immunohistochemistry [1]. On fine-needle aspiration biopsy (FNAB) of the breast, the characteristic cytological features include multiple cohesive morules, angulated papillaroid clusters with a jigsaw puzzle-like appearance, and singly scattered tumor cells [3].

Reversed cellular polarity - a hallmark of IMPC - was first identified by Luna-Moré et al. using electron microscopy [4]. This phenomenon is characterized by the aberrant localization of secretions and blebs on the basal surface of tumor cells, along with MUC1 (Mucin1) (EMA) immunostain expression, which distinguishes IMPC from other breast carcinoma subtypes. MUC1 promotes the separation of tumor cells from the extracellular matrix, thereby enhancing cellular dispersion and facilitating early LNM. The molecules sLex and CXCR4 are involved in mechanisms of tumor immune evasion. Annexin A2 (ANXA2), a cellular polarity protein essential for lumen formation, when compromised, leads to increased apoptosis and reduced anti-apoptotic activity. Furthermore, Galectin-3, plakoglobin, LEF1, and ARID1A have all been implicated in the progression of IMPC [2].

FNAB has long been an effective method for the pathological assessment of primary breast carcinoma. However, in recent years, core needle biopsy (CNB) has become the preferred diagnostic approach. This transition is largely due to CNB’s superior ability to accurately differentiate invasive from in situ disease. Moreover, CNB facilitates standardized evaluation of hormone receptor status and human epidermal growth factor receptor 2 (HER2), both critical for guiding targeted therapies. Compared with FNAB, CNB offers lower inter-observer variability, higher sensitivity (85-100% versus 35-95%), greater specificity (86-100% versus 48-100%), and improved overall diagnostic accuracy (84% versus 72%) [5].

This case series aimed to evaluate the utility of FNAB - a rapid, reliable, and cost-effective technique - for assessing peripheral metastatic sites in suspected breast malignancies, an area that remains underexplored, particularly in terms of its predictive value for diagnosing the rare and aggressive IMPC subtype.

## Case presentation

Cytological samples from metastatic sites, displaying angulated, avascular, jigsaw-puzzle-like papillaroid clusters and/or morules/cell ball-like clusters, obtained by FNAB in suspected breast cancer patients performed between 2022 and 2024, with available histopathological diagnoses and clinical details available, were included.

Radiologists or pathologists performed all aspirations. Using a single or repeated passes in the mass while maintaining negative pressure, the aspirates were extracted using a disposable 10-mL syringe and a disposable 22-gauge needle. Standardized amount of material was placed on each slide for staining to ensure consistency across all samples. Romanowsky and Papanicolaou stains, with hematoxylin and eosin (H&E), were applied to the smears. Cell block preparation and immunocytochemistry were not performed for any of the samples, as per routine laboratory protocol; cell block preparation is not done routinely in cases where initial smears are deemed sufficient for diagnosis.

Quantitative scoring by conventional microscopy was performed independently by two pathologists (O.G., R.G.) for cellularity, tumor cell arrangement, cytoplasmic vacuoles, and background mucin. Cytological grading was done as described by Robinson et al. [6].

Cellularity is the percentage of nuclei relative to the total smear area, which may contain background elements such as mucin, necrosis, red blood cells, lymphoid cells, and/or debris.

Tumor cell arrangement (100%) was quantified for (a) angulated avascular papillaroid cluster, (b) morule or cell ball-like cluster, (c) sheets, (d) acini, (e) loosely cohesive cluster (other than a, b, c, d), and isolated malignant cells.

Individual tumor cells were quantified for cytoplasmic vacuoles.

The mean values for each quantitative cytological parameter were determined by summing the scores from all slides per case and incorporating the assessments of the pathologist. Values less than 25% were classified as low, 25-49% as moderate, and ≥50% as high.

The histopathological diagnoses followed the latest WHO guidelines [1].

Case 1

A 57-year-old female patient presented with a 5-cm lump in the left breast and left axillary lymphadenopathy, persisting for three years. FNAB of the left axillary lymph node yielded five smears, revealing low cellularity (10%) and abundant background mucin (70%). The atypical cells were grade 1 and predominantly formed loosely cohesive clusters (25%), with additional angulated, avascular papillaroid clusters (20%) and morule or cell ball-like clusters (15%) with reversal of cell polarity, sheets (15%), and acini (10%). Cytoplasmic vacuoles were observed in 20% of these atypical cells. Subsequent left mastectomy identified an IMPC component in 30% of the tumor cells, leading to a diagnosis of mixed mucinous carcinoma with IMPC grade 1.

Case 2

A 42-year-old female patient presented with a right breast lump of one year’s duration, measuring 3 cm on examination, accompanied by right axillary lymphadenopathy. FNAB of the right axillary lymph node was performed, yielding seven smears demonstrating 30% cellularity with moderate background mucin (40%). The atypical cells were grade 2 and predominantly formed angulated, avascular papillaroid clusters (50%), followed by morule or cell ball-like clusters (15%) with reversal of cell polarity, with equal proportions of sheets (10%) and loosely cohesive clusters (10%), and a smaller proportion of acini (5%). Cytoplasmic vacuoles were observed in 10% of these atypical cells. Subsequent right mastectomy revealed an IMPC component in 60% of the tumor cells, leading to a diagnosis of IBC of no special type (IBC-NST) with mucin production and IMPC grade 2.

Case 3

A 58-year-old female patient initially presented with a right breast lump of three years’ duration, measuring 4 cm on examination, accompanied by right axillary lymphadenopathy. FNAB of the right axillary lymph node was performed, yielding two smears that demonstrated 40% cellularity with moderate background mucin (45%). The atypical cells were grade 2 and predominantly formed morule or cell ball-like clusters (40%), followed by angulated, avascular papillaroid clusters (20%) with reversal of cell polarity, sheets (15%), acini and isolated cells (10% each), and loosely cohesive clusters (5%). Cytoplasmic vacuoles were observed in 20% of these atypical cells. Subsequent right modified radical mastectomy revealed an IMPC component in 60% of the tumor cells, leading to a diagnosis of IBC-NST with mucin production and IMPC grade 2. She received systemic therapy as per standard protocols; however, after 20 months, she developed a left breast lump with left axillary lymphadenopathy. FNAB from the left axillary lymph node yielded four smears, which showed 30% cellularity with low background mucin (5%). The atypical cells were of grade 3, predominantly forming morule or cell ball-like clusters (35%), closely followed by angulated, avascular papillaroid clusters (30%) with reversal of cell polarity, loosely cohesive clusters (15%), sheets (10%), and acini and isolated cells (5% each). Cytoplasmic vacuoles were noted in 5% of these atypical cells. Left mastectomy revealed an IMPC component in 60% of the tumor cells, leading to a final diagnosis of IBC-NST with mucin production and IMPC grade 3.

Case 4

An 83-year-old man presented with two cutaneous nodules, one on the right arm and the other on the back. FNAB was performed on both nodules, with four smears prepared from each. Both samples showed highly cellular smears (50% cellularity), cytological grade 3, with a predominance of morule or cell ball-like clusters (40% and 30%), closely followed by angulated, avascular papillaroid clusters (30% in both) with reversal of cell polarity, equal proportions of loosely cohesive clusters (10%), and isolated malignant cells (15%). Background mucin was scanty (absent and 5%), and cytoplasmic mucin was present in 30% of atypical cells. Surgical excision of both skin lesions confirmed metastatic IMPC with more than 90% IMPC component. On review, he was found to be a known case of IMPC grade 3 of the right breast with metastatic lymph nodes, diagnosed six years earlier with 95% IMPC component on histopathology at the primary site.

The findings from all the samples in these cases are summarized in Table 1.

**Table 1:** Clinicopathological features of four cases and cytological findings of their six samples FNAB, fine-needle aspiration biopsy; IBC-NST, invasive breast carcinoma of no special type; IMPC, invasive micropapillary carcinoma

Case No. (Sample No.)	1	2	3 (1)	3 (2)	4 (1)	4 (2)
Age (in years)	57	42	58		83	
Sex	Female	Female	Female		Male	
Laterality of breast malignancy	Left	Right	Right	Left	Right	
IMPC proportion (in primary site)	30%	60%	60%		More than 90%	
Site(s) of FNAB	Left axillary lymph node	Right axillary lymph node	Right axillary lymph node	Left axillary lymph node	Right arm nodule	Back nodule
Histopathology diagnosis of primary breast carcinoma [1]	Mixed mucinous carcinoma with IMPC grade 1	Mixed IBC-NST with mucin production and IMPC grade 2	Mixed IBC-NST with mucin production and IMPC grade 2	Mixed IBC-NST with mucin production and IMPC grade 3	IMPC grade 3	
FNAB findings						
No. of smears	5	7	2	4	4	4
Cellularity (%)	10	30	40	30	50	50
Cytological grade [6]	1	2	2	3	3	3
(a) Angulated, avascular papillaroid cluster (%)	20	50	20	30	30	30
(b) Morule or cell ball (%)	15	15	40	35	40	30
(c) Sheet (%)	15	10	15	10	5	5
(d) Acini (%)	10	5	10	5	5	5
(e) Loosely cohesive clusters (other than a, b, c, d) (%)	25	10	5	15	10	15
(f) Isolated malignant cells (%)	15	10	10	5	10	15
Background mucin (%)	70	40	45	5	0	5
Cytoplasmic vacuoles (%)	20	10	20	5	20	30

## Discussion

The cases include one male, 83 years old, and three females, 42, 57, and 58 years old, respectively, with a mean age of 60 years. Male breast cancer is rare, representing less than 1% of all male malignancies and only 1% of all breast cancer cases [1]. Case 4 represents one such rare event. The median age for diagnosing IMPC in males is 69.8 years, slightly older than that observed in female patients [7], much older in our case 4.

The right breast was involved in three cases (2, 3, and 4) and the left breast in one (case 1); however, one female later developed metachronous left breast malignancy as well (case 3). All individuals underwent mastectomy with axillary lymphadenectomy, and the male patient additionally had excision of a metastatic skin nodule (case 4). IMPC is aggressive due to its high propensity for LVI, axillary lymph node, and other metastatic site involvement [2], as observed in all our cases. Male breast cancer is typically diagnosed at more advanced ages and stages, which negatively impacts treatment outcomes and survival rates [7], as seen in case 4.

Histologically, IMPC is characterized by hollow or morule-like aggregates and angulated papillaroid clusters of cuboidal to columnar neoplastic cells, devoid of fibrovascular core and immersed in clear, spongy stroma [1]. All the four cases had IMPC components diagnosed histopathologically in the breast, ranging from 30% to 95%, with 60% in two cases. As per the latest guidelines [1], the histopathological diagnoses were one case of mixed mucinous carcinoma with IMPC grade 1 (case 1), two cases of mixed IBC-NST with mucin production and IMPC grades 2 and 3 (cases 2 and 3), and one case of pure IMPC grade 3, in an elderly male (case 4).

FNAB was performed on two axillary lymph nodes on each side (left and right) (cases 1, 2, and 3), as well as on two skin nodules, one located on the right arm and the other on the back (case 4).

**Figure 1:**
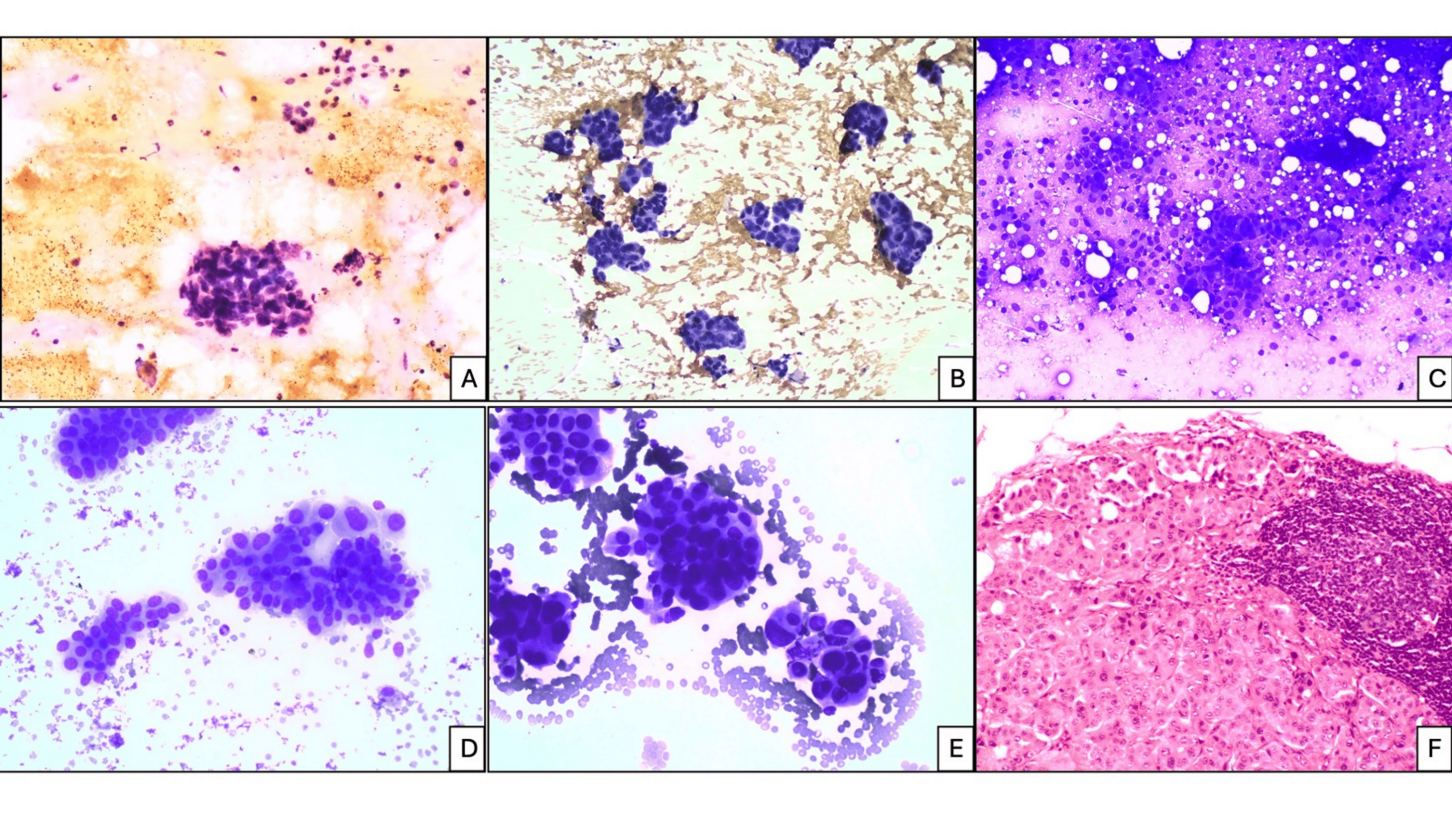
FNAB cytology. Cellularity: (A) low, and cytologic grade 1 (H&E stain, x100), (B) moderate (Papanicolaou stain, x100), (C) high (Romanowsky stain, x40). Cytologic grade: (D) 2, and mild loosely cohesive clusters (Romanowsky stain, x200), (E) 3 (Romanowsky stain, x200), (F) lymph node HPE showing metastasis of IMPC (H&E stain, x100). FNAB, fine-needle aspiration biopsy; H&E, hematoxylin and eosin; HPE, histopathological examination

**Figure 2:**
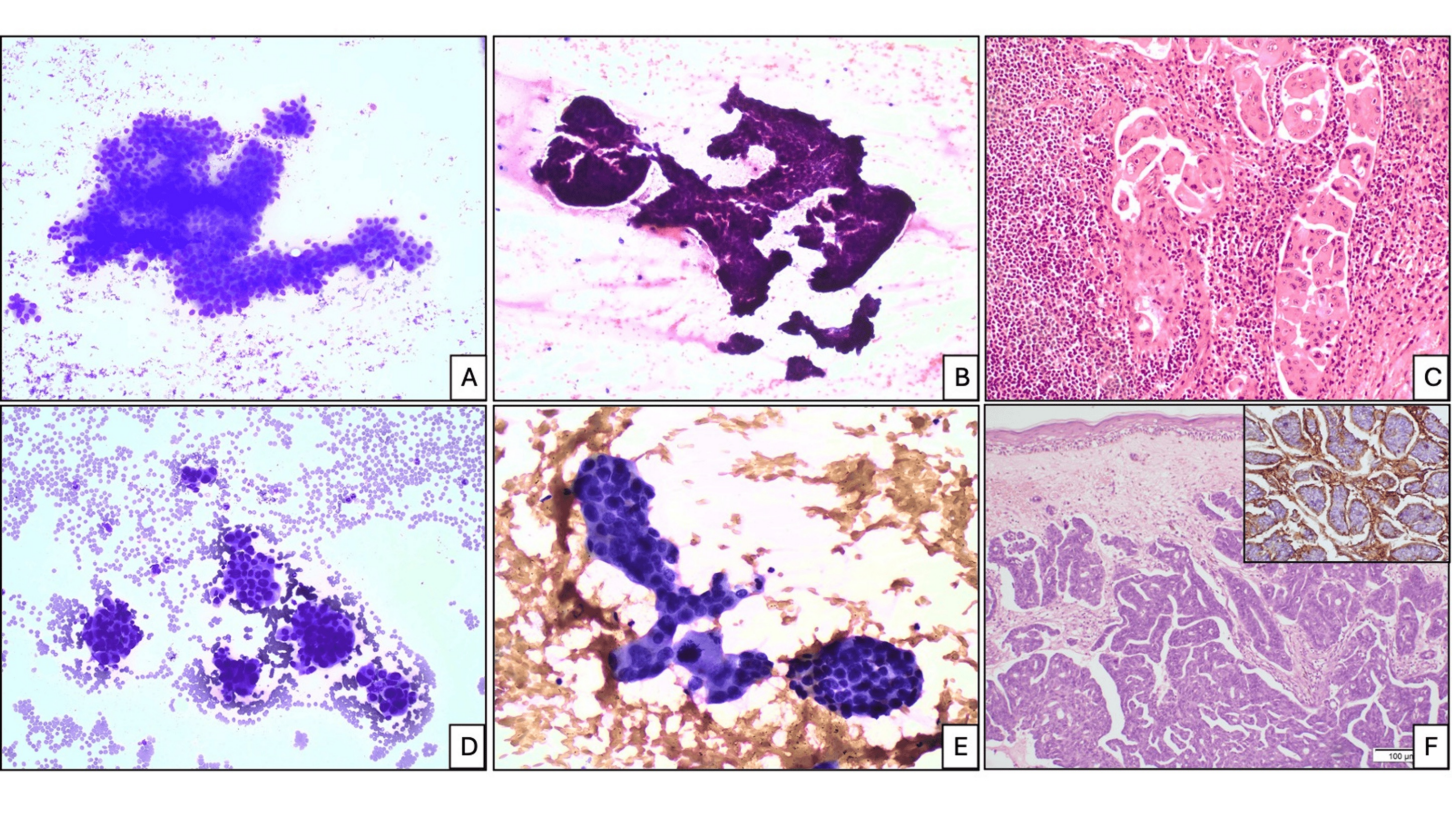
FNAB cytology. (A) Angulated papillaroid cluster (Romanowsky stain, x100), (B) zig-saw puzzle-like angulated papillaroid clusters (H&E stain, x100), (C) corresponding HPE from a metastatic lymph node (H&E stain, x200). FNAB cytology: (D) Morule or cell ball-like clusters (Romanowsky stain, x100), (E) (Papanicolaou stain, x200), (F) corresponding HPE of right arm metastasis (H&E stain, x100). Inset: EMA immunostaining highlighting reversal of cellular polarity with basal surface positivity. EMA, epithelial membrane antigen; FNAB, fine-needle aspiration biopsy; H&E, hematoxylin and eosin; HPE, histopathological examination

**Figure 3:**
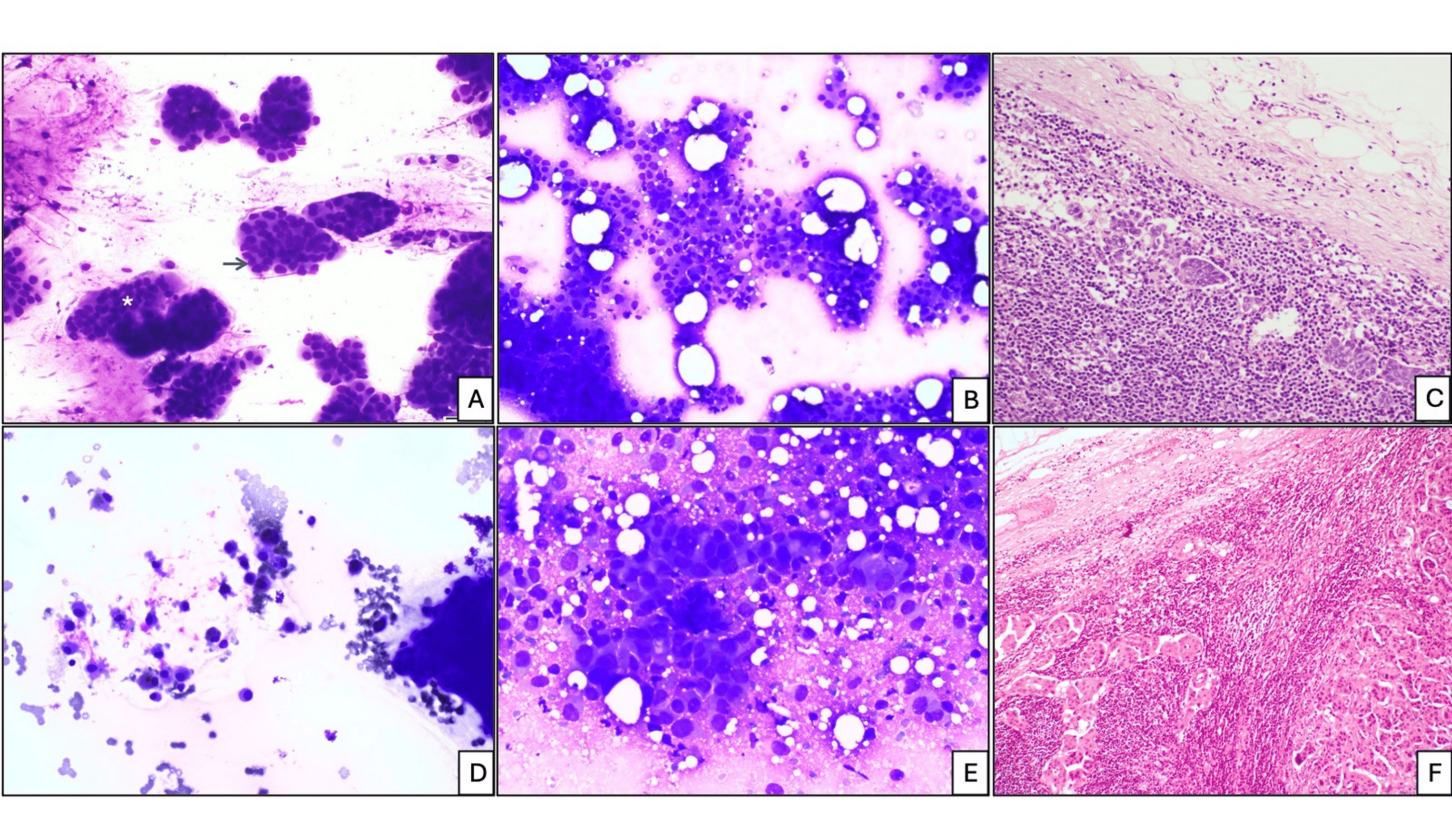
FNAB cytology. Sheets: (A) low (white asterisk), acini (blue arrow) (Romanowsky stain, x200), (B) loosely cohesive clusters: moderate (Romanowsky stain, x100), (C) corresponding HPE from a metastatic lymph node (H&E stain, x100). Isolated malignant cells: (D) low (Romanowsky stain, x100), (E) low (Romanowsky stain, x400), (F) corresponding HPE from a metastatic lymph node (H&E stain, x200). FNAB, fine-needle aspiration biopsy; H&E, hematoxylin and eosin; HPE, histopathological examination

**Figure 4:**
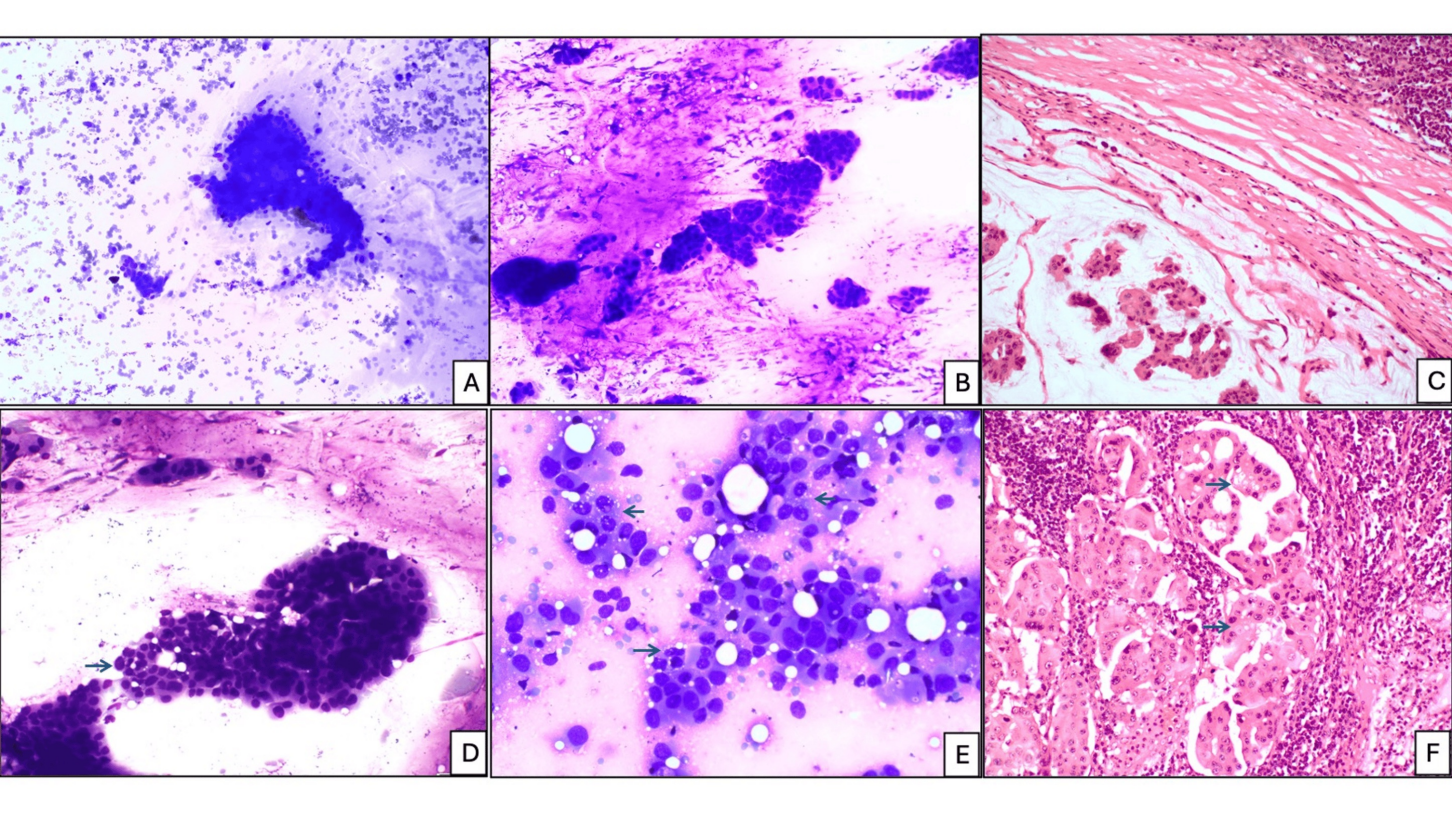
FNAB cytology. Background mucin (A) moderate (Romanowsky stain, x200), (B) high (Romanowsky stain, x100), (C) corresponding HPE of metastatic lymph node with mucin (H&E stain, x200). Cytoplasmic vacuoles: (D) low (blue arrow) (Romanowsky stain, x200), (E) moderate (blue arrows) and moderate loosely cohesive clusters (Romanowsky stain, x200), (F) corresponding HPE of lymph node metastasis showing cytoplasmic vacuoles in tumor cells (blue arrow) (H&E stain, x200). FNAB, fine-needle aspiration biopsy; H&E, hematoxylin and eosin; HPE, histopathological examination

A spectrum of cytomorphological appearances was observed including variable cellularity, identified as low (1), moderate (3), high (2) (Figures 1A-1C); cytological grades 1 (1), 2 (2), 3 (3) (Figures 1A, 1D, 1E); angulated, avascular papillaroid clusters, identified as low (2), moderate (3), high (1), (Figures 2A, 2B); morule/cell ball-like clusters, identified as low (2), moderate (4) (Figures 2D, 2E); sheets, identified as low (6) (Figure 3A); acini, identified as low (6) (Figure 3A); loosely cohesive clusters, identified as low (5), moderate (1) (Figures 3B, 4E); isolated malignant cells, identified as low (6) (Figures 3D, 3E); background mucin, identified as absent (1), low (2), moderate (2), high (1) (Figures 4A, 4B); and cytoplasmic vacuoles, identified as low (5) and moderate (1) (Figures 4D, 4E).

**Figure 5:**
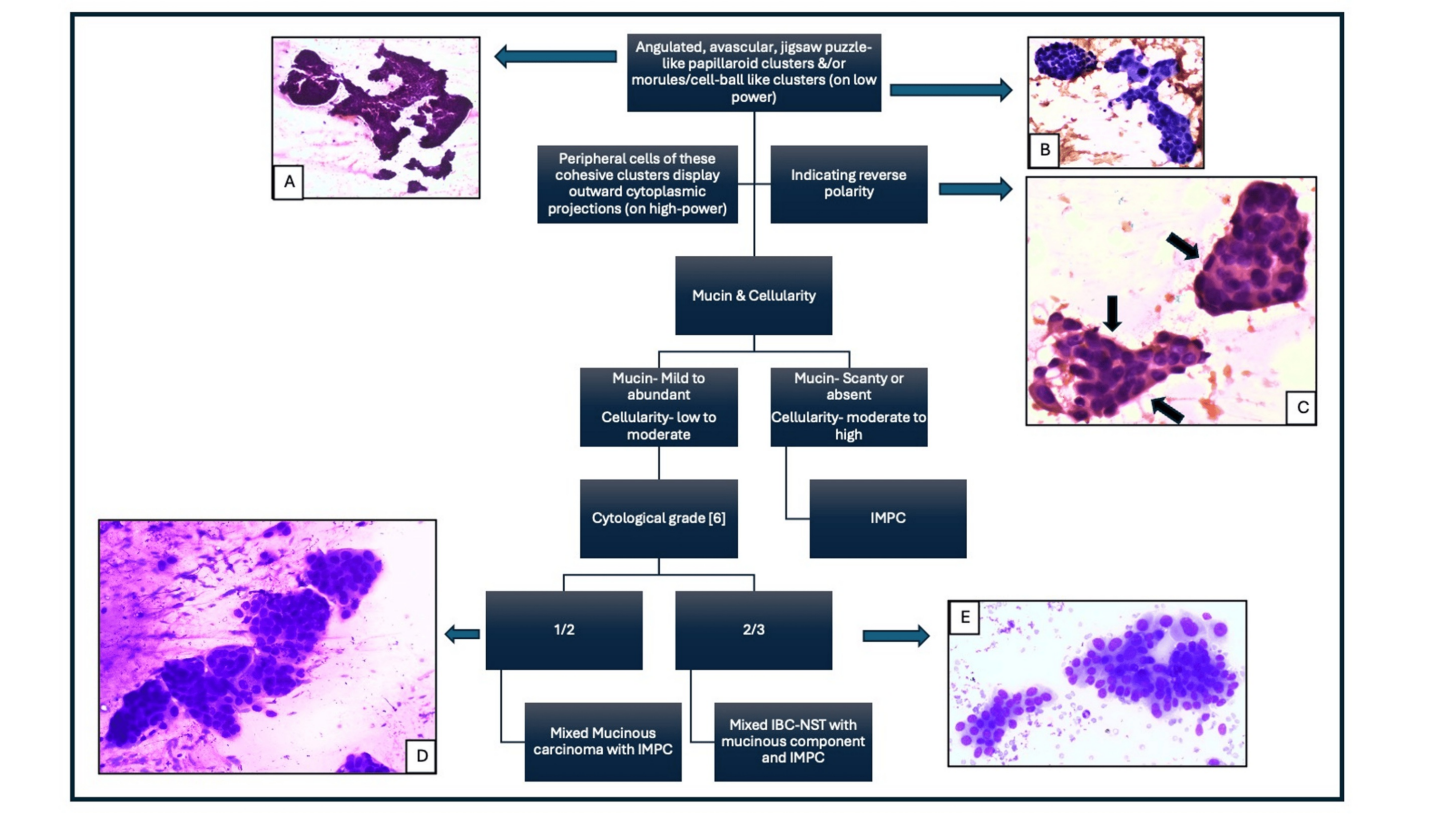
Algorithmic approach to close differential diagnoses of micropapillary components at the metastatic site in suspected breast cancer. (A) From Figure 2B and (B) from Figure 2E. (C) Oval to angulated, avascular papillaroid clusters. Black arrows indicate cytoplasmic projections in the peripheral cells, indicating reverse polarity (Papanicolaou stain, x400). (D) From Figure 4D and (E) from Figure 1D.

The cytological findings in our series indicate that increased cellularity, higher cytological grade, angulated papillaroid clusters, and morule or cell ball-like structures with reversal of cell polarity (indicated by secretions and blebs at the outer cell membrane of these clusters) (Figures 5A-5C), loosely cohesive clusters, and isolated malignant cells - combined with a reduction in background mucin at metastatic sites on FNAB - correlate with a higher proportion of IMPC in the primary breast lesion, which is associated with a poorer prognosis. In pure IMPC, the tumor cells form similar angulated, cohesive clusters of neoplastic cells, lacking a fibrovascular center [3].

The cytological differential diagnosis of IMPC includes other primary breast carcinomas such as invasive papillary carcinoma (IPC), mucinous carcinoma, mixed mucinous carcinoma with IMPC, IBC-NST with mucinous component, and IMPC and metastatic tumors. IPC shows complex and slender papillae with thin fibrovascular cores, increased intact and dissociated tall columnar cells displaying variable nuclear atypia, and the absence of myoepithelial cells [3]. Cases 2, 3, and 4 exhibited moderately pleomorphic cells arranged in compact, angulated, jig-saw-like papillaroid clusters, lacking both fibrovascular cores and myoepithelial cells. Pure mucinous carcinoma is characterized by moderate to abundant mucin in the background, with monomorphic tumor cells arranged in singly scattered, tubules, cribriform, or loosely cohesive, rounded patterns without any polarity reversal [3], which was lacking in our cases. A simple algorithm presented in Figure 5 will help in differentiating close mimickers of IMPC of the breast.

A study by Oz et al. [8] demonstrated that pure IMPC, defined as tumors with more than 90% IMPC component, and mixed IMPC exhibit significant differences in both molecular characteristics and prognostic outcomes. These findings highlight the critical importance of accurately distinguishing between pure and mixed IMPC subtypes for appropriate clinical assessment and management.

A study conducted by Elzohery et al. [9] demonstrated that patients with IMPC exhibited a significantly lower rate of breast-conserving surgery (26% compared to 37.8%), a higher incidence of modified radical mastectomy (49.3% versus 46%), and an increased requirement for systemic therapy (77.5% versus 41%) when compared to those with IBC of no special type (IBC-NST). These findings highlight the critical importance of accurately identifying IMPC, even at metastatic sites, to guide optimal management strategies.

On occasion, the cytological differential diagnosis may encompass non-mammary malignancies, such as papillary serous carcinoma of the female genital tract, as well as micropapillary carcinoma of the lung and urinary bladder. Given the markedly distinct therapeutic approaches required for these entities compared to breast carcinoma, they must be considered and systematically excluded through comprehensive clinical and radiological correlation. In instances where diagnostic ambiguity persists, ancillary techniques, including cell block preparation and/or immunohistochemical analysis, should be employed to facilitate definitive diagnosis. Serous carcinomas typically exhibit almost diffuse, marked nuclear pleomorphism, frequent psammomatous calcifications, atypical mitoses, and positive immunostaining for PAX8, WT1, and p53 on cell block [10-12]. Metastasis from the lung can be indicated by focal squamoid differentiation and confirmed by positive TTF1 and Napsin A and negative p40, GATA-3, and mammaglobin immunostains on cell block. Metastasis from the urinary bladder can be suspected by abundant eosinophilic cytoplasm of transitional cell differentiation and confirmed by positive GATA3, CK20, and negative mammaglobin immunostains on cell block [10-12].

## Conclusions

Increased cellularity, higher cytological grade, jig-saw-pattern-like avascular angulated papillaroid clusters, and morule or cell ball-like structures with reversal of cell polarity (indicated by secretions and blebs at the outer cell membrane of these clusters), loosely cohesive clusters, and isolated malignant cells - combined with a reduction in background mucin at metastatic sites on FNAB - correlate with a higher proportion of IMPC in the primary breast lesion, which is associated with a poorer prognosis. FNAB, being a rapid, simple, and minimally invasive technique, is highly valuable for assessing peripheral metastatic sites. It not only aids in detecting this rare and aggressive breast cancer subtype but also provides insight into its proportion within the primary tumor, thereby facilitating optimal management strategies. Larger multicenter studies are recommended to validate the findings of this case series.
